# Ex-Vivo Skin Explant Culture Is a Model for TSLP-Mediated Skin Barrier Immunity

**DOI:** 10.3390/life11111237

**Published:** 2021-11-16

**Authors:** Thomas Bauer, Daniela Gubi, Jörg Klufa, Philipp Novoszel, Martin Holcmann, Maria Sibilia

**Affiliations:** Department of Medicine I, Institute of Cancer Research, Medical University of Vienna and Comprehensive Cancer Center, 1090 Vienna, Austria; daniela.gubi@gmx.net (D.G.); joerg.klufa@boehringer-ingelheim.com (J.K.); philipp.novoszel@meduniwien.ac.at (P.N.); martin.holcmann@meduniwien.ac.at (M.H.)

**Keywords:** skin, EGFR, Langerhans cell, atopic inflammation, TSLP, barrier immunity, JNK-signalling

## Abstract

The skin is the outermost barrier protecting the body from pathogenic invasion and environmental insults. Its breakdown initiates the start of skin inflammation. The epidermal growth factor (EGFR) on keratinocytes protects this barrier, and its dysfunction leads to atopic dermatitis-like skin disease. One of the initial cytokines expressed upon skin barrier breach and during atopic dermatitis is TSLP. Here, we describe the expression and secretion of TSLP during EGFR inhibition and present an ex-vivo model, which mimics the early events after barrier insult. Skin explants floated on culture medium at 32 °C released TSLP in parallel to the activation of the resident Langerhans cell network. We could further show the up-regulation and activation of the AP-1 family of transcription factors during atopic-like skin inflammation and its involvement in TSLP production from the skin explant cultures. Inhibition of the c-Jun N-terminal kinase pathway led to a dose-dependent blunting of TSLP release. These data indicate the involvement of AP-1 during the early stages of atopic-like skin inflammation and highlight a novel therapeutic approach by targeting it. Therefore, skin explant cultures mimic the early events during skin barrier immunity and provide a suitable model to test therapeutic intervention.

## 1. Introduction

The mammalian skin is a complex organ evolved to protect the body from external environmental and pathogenic affronts [1]. When the physical barrier is breached, keratinocytes (KC), the primary cell type of the epidermis, and Langerhans cells (LC), the outermost sentinels of the immune system, are among the first responders [2]. LCs act as professional antigen-presenting cells and pick up antigens with which they migrate to the skin-draining lymph nodes to activate the adaptive arm of the immune system [2]. KCs react to threats by producing and releasing pro-inflammatory cytokines and chemokines, thereby initiating immune cell recruitment and regulating the immune response [3].

Thymic stromal lymphopoietin (TSLP) is amongst the first cytokines produced and released primarily by epithelial cells at barrier surfaces, such as the skin and the lung, in response to danger signals [4]. TSLP starts the inflammatory cascade by activating the LCs surrounding the KCs. The activated LCs and dendritic cells (DCs) then initiate adaptive T-cell responses. TSLP is a well-known initiator of allergic T-helper 2 (Th2) responses, which are hallmarks of atopic dermatitis (AD) [5].

AD or eczema is mainly characterized by itchy, dry, red, and cracked skin. Secondary skin infections by *Staphylococcus aureus* usually worsen the disease. Its cause is unknown, but it is believed that genetic predispositions involving the immune system or skin barrier dysfunctions are the main drivers [6].

The AP-1 family of transcription factors has been shown to be up-regulated in lesions of AD patients [7]. These basic leucine zipper transcription factors are crucial for skin homeostasis. Opposite to AD, the epidermal lack of c-Jun/JunB induces a psoriatic phenotype in mice [8]. AP-1 regulates gene expression upon various stimuli, including cellular stress, cytokines, and growth factors. In DCs, c-Jun is crucial for cytokine and chemokine expression [9].

We have recently shown that the lack of EGFR in the skin (EGFR deletion using the keratin 5 promoter, hereafter referred to as EGFR^Δep^ mice) leads to a breakdown of the epidermal barrier upon hair eruption. The transcriptional profile of the resulting skin disease closely resembles human AD, including the phenotypic hallmarks like itchy and dry skin, *S. aureus* superinfections, and a microbiota-independent induction of TSLP [10]. EGFR inhibition (EGFR-I) is a well-established, targeted anti-cancer therapy used for many solid tumors [11]. Its cutaneous adverse events reflect the importance of EGFR during skin homeostasis [3,12].

In this study, we used the EGFR^Δep^ mouse as an AD model to gain more insight into epidermal TSLP secretion. In parallel, we developed an ex-vivo skin explant model that mimics TSLP expression upon barrier insult and opens up a way to explore different treatment options. Therefore, we aim for the identification and development of practicable therapeutic options to ameliorate Th2 driven atopic skin diseases and adverse events during EGFR-targeted anti-cancer therapy.

## 2. Materials and Methods

### 2.1. Mice

EGFR^Δep^ and c-Jun^Δep^ mice were generated as previously described [3,9]. All mice in this study are in the C57BL/6 background and were bred and maintained in the facilities of the Medical University of Vienna in accordance with institutional policies and federal guidelines. All mice had access to food and water ad libitum. Animal experimental procedures were approved by the Animal Experimental Ethics Committee of the Medical University of Vienna and the Austrian Federal Ministry of Science and Research (animal license numbers: GZ 66.009/124-BrGT/2003; GZ 66.009/109-BrGT/2003; GZ BMWF-66.009/0073-II/10b/2010; GZ BMWF-66.009/0074-II/10b/2010; GZ BMWFW-66.009/0200-WF/II/3b/2014; GZ BMWFW-66.009/0199-WF/II/3b/2014).

### 2.2. Serum Isolation

Blood was taken from mice of different ages and treatments via heart puncture. The blood rested between 30 min to 3 h to allow it to clot. The supernatant was collected after centrifugation (400× *g*, 15 min), and the serum was shock frozen in liquid nitrogen and stored at −20 °C for further use.

### 2.3. Epidermal Ear Sheets

To separate epidermis from dermis, mouse ear splits were floated at 37 °C with dermal sides facing down on 3.5% ammonium thiocyanate for 25 min and subsequently fixed with 4% PFA for 30 min on room temperature and further subjected to immunofluorescence staining.

### 2.4. Histological Analysis and Immunofluorescence Microscopy

For immunofluorescence staining of cryo-sections, dorsal skin was embedded in OCT (Sakura, fisher scientific, Schwerte, Germany), immediately frozen, cut into 5-µm sections, and post-fixed with 4% PFA (Roth, Krems, Austria) for 30 min at room temperature. Subsequently, skin sections or epidermal sheets were blocked with 5% goat serum (Merck, Darmstadt, Germany) and 2% BSA TBS-T (Merck, Darmstadt, Germany) for 1 h and incubated with primary antibodies diluted in 5% goat serum and 2% BSA TBS-T at 4 °C overnight. Subsequently, slides were rinsed and incubated with an appropriate secondary antibody and Hoechst (Sigma-Aldrich, St. Louis, MO, UAS) for 2 h in a dark humidified slide chamber. Tissue sections were mounted, and pictures were taken using a Nikon eclipse 80i microscope (Nikon, Tokyo, Japan). Antibodies used are listed in Table 1.

### 2.5. Epidermal Single Cell Suspension and Flow Cytometry Analysis

Mice were euthanized, and mouse ears were split into dorsal and ventral side and placed on 0.8% trypsin (Gibco, Thermo Fisher Scientific, Waltham, MA, USA) for 45 min at 37 °C in order to allow the separation of epidermis and dermis. The epidermis was cut into pieces, further digested in 250 µg/mL DNase I (Merck, Darmstadt, Germany) for 30 min at 37 °C, washed, and filtered through a 70-µm cell strainer.

Single-cell suspensions were subsequently blocked with FC-block (BD Pharmingen, Franklin Lakes, NJ, USA) and stained with indicated fluorescently labelled antibodies (Bio-Legend, San Diego, CA, USA) at 4 °C for 30 min. Antibodies used are listed in Table 1. Prior to flow cytometry analysis, SYTOX™ Blue Dead Cell Stain (Invitrogen, Thermo Fisher Scientific, Waltham, MA, USA) was added according to the manufacturer’s recommendations to discriminate dead from living cells. Cells were recorded using an LSR-II flow cytometer (BD Biosciences, Franklin Lakes, NJ, USA) and analysed using FlowJo software 7.6.4. (Becton Dickinson, Ashland, OR, USA)

### 2.6. Skin Explant Cultures

Fresh mouse ears were split, cut into 1-cm^2^ pieces, and floated dermal side down on 1 mL RPMI medium containing 10% FCS and Penicillin/Streptavidin in a 24-well plate at either RT (20–24 °C) or 32 °C for 24 h. Skin samples were either treated with tacrolimus (Prograf, Astellas Pharma, Tokyo, Japan) or SP600125 (Merck, Darmstadt, Germany) in the culture medium. Concentrations were used as indicated in the figure legends. After 24 h, media was taken, snap frozen in liquid nitrogen, and stored at −20 °C until further use. Media was analysed for cytokine expression via ELISA.

### 2.7. ELISA

Quantification of mouse TSLP concentration in serum, total protein from skin biopsies, and media from ear explant cultures was performed by enzyme-linked immunosorbent assay (ELISA) according to the DuoSet ELISA Development kit protocol or Quantikine ELISA Development kit protocol (R&D Systems). Microplate reader (Tecan infinite 200 PRO, Hombrechtikon, Switzerland) measured absorbance at 450 nm, with the correction wavelength set at 540 or 570 nm.

### 2.8. Skin Protein Isolation

Skin biopsies from the back or ear skin were taken and immediately snap-frozen and stored at −80 °C for further use. For homogenization, skin biopsies were added to RIPA lysis buffer, supplemented with Protease Inhibitor Cocktail (Roche, Basel, Switzerland), and homogenized in Precellys tubes containing ceramic beads (VWR, Radnor, PA, USA) using a Precellys 24 homogenizer (Bertin; 2 × 30 s at 6000 rpm followed by 30 s on ice after each cycle). Skin lysates were then transferred to Eppendorf tubes and centrifuged at 14,000× *g* for 15 min at 4 °C to remove cell debris. The supernatant was transferred to a new Eppendorf tube and subsequently snap-frozen and stored at −80 °C. For quantification of serum protein, 50 µL of murine serum was used.

### 2.9. Statistical Methods

All experiments presented in the figures were repeated at least two independent times. Statistical comparisons were performed using GraphPad Prism 5.02 software (GraphPad, San Diego, CA, USA). Data are represented as means +/− SEM. N describes the number of biological replicates. Statistical significance was determined using Student’s unpaired two-tailed *t*-test for comparison of two groups and one-way ANOVA followed by Tukey’s post-hoc test for multiple comparison. *p* < 0.05 was taken to be statistically significant, and *p*-values are represented with asterisks in the figures. (* *p* < 0.05; ** *p* < 0.005; *** *p* < 0.0005).

## 3. Results

### 3.1. TSLP Is Induced and Released from EGFR^∆^^ep^ Skin In Vivo and Ex Vivo

**Figure 1:**
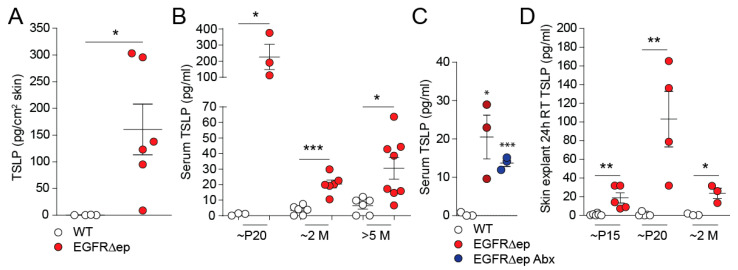
TSLP is induced and released from EGFR∆ep skin in vivo and ex vivo. (**A**) Protein levels of TSLP from WT and EGFR∆ep mice per 1 cm^2^ total skin. (**B**) Protein levels of TSLP from WT and EGFR∆ep mice at various time points (*p* refers to postnatal day, and M refers to month-old mice), as measured in the serum. (**C**) 5M-old EGFR∆ep mice were treated with the antibiotic cefazolin (0.5 g/L; Abx) in drinking water for 1M, and serum TSLP were measured by Elisa. (**D**) Protein levels of TSLP from WT and EGFR∆ep mice at various time points (*p*, postnatal day; M, month), as measured from culture supernatants of ear skin explant cultures cultivated for 24 h at room temperature (RT). Data represent means ± SEM; * *p* < 0.05; ** *p* < 0.01; *** *p* < 0.001; Each dot represents data from one individual mouse or skin explant.

High levels of TSLP are found in lesional skin of patients with allergic forms of dermatitis [4]. We recently developed a mouse model of atopic dermatitis-like skin inflammation where we could also find TSLP expressed in the skin (Figure 1A) [10]. TSLP from this mouse dermatitis model was also detectable in the serum of EGFR^∆ep^ mice, with a peak expression around postnatal day 20, which represents two weeks after the start of the skin inflammation (p20, Figure 1B). In order to rule out bacterial involvement of TSLP secretion in EGFR^∆ep^ mice, we treated five-month-old mice with antibiotics (Abx) for one month. Remarkably, TSLP expression was still detectable in levels comparable to untreated EGFR^∆ep^ mice (Figure 1C).

In order to establish a model of TSLP secretion from the skin, which mirrors the in-vivo situation, we floated ear halves or back skin from EGFR^∆ep^ mice on cell culture-media overnight at room temperature (RT, 20–24 °C). TSLP secretion could be readily detected in the culture medium and was found to follow the trend of the in-vivo serum expression from Figure 1B, which peaked in three-week-old EGFR^∆ep^ mice (Figure 1C). After the peak of expression at P20 (Postnatal day 20), TSLP levels remained relatively high in EGFR^∆ep^ mice compared to WT (Figure 1B,C).

These data indicate that TSLP expression is secreted from inflamed skin in vivo and that this can be mimicked using skin explants floated on RT for 24 h ex vivo.

### 3.2. TSLP Expression from Skin Explants Cultured at 32 °C Represents an Ex-Vivo Model for Skin Barrier Immunity

**Figure 2:**
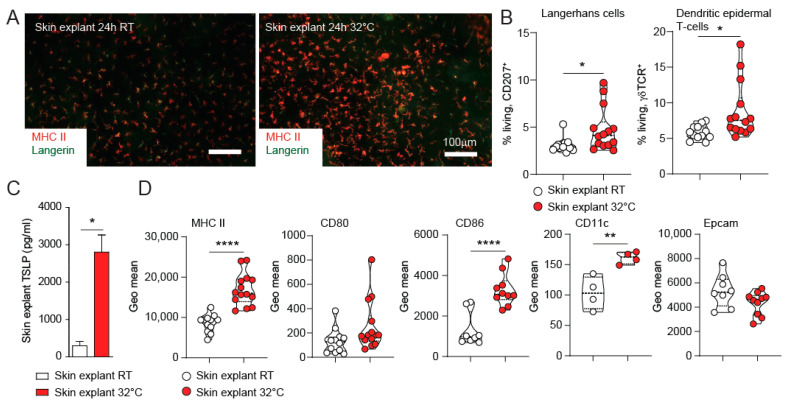
TSLP expression of skin explants cultured at 32 °C represents an ex-vivo model for skin barrier immunity. (**A**) Ears from 1–2-month-old WT mice were separated and floated dermal side down for 24h on medium at room temperature and 32 °C, respectively. Epidermal sheets were isolated and stained for Langerin (CD207) and MHC II (I-A/I-E) to detect LCs and their activation status. Pictures are representatives from at least three independent experiments. (**B**) Epidermal cell suspensions from these cultures were FACS analysed for CD207 and gdT-cell receptor (gdTCR) to identify LCs and DETCs. (**C**) Culture supernatant was collected from these cultures, and TSLP expression was detected by ELISA. Data are from at least three independent experiments. (**D**) Epidermal cell suspensions from these cultures were FACS analysed. LCs were gated using CD207, and the MFI for the indicated markers is shown. Data represent means ± SEM; * *p* < 0.05; ** *p* < 0.01; **** *p* < 0.0001; each dot represents data from one individual skin explant.

We show here that TSLP is secreted from ear skin explant cultures from EGFR^Δep^ mice ex vivo when cultured at RT (Figure 1C). Ear skin explants from WT mice did not show TSLP secretion when cultured at RT for 24 h. However, culturing skin explants from WT mice at 32 °C for 24 h induced morphological changes together with MHC II up-regulation on Langerhans cells (LCs, Figure 2A). Using FACS analysis, we can show that the local epidermal immune cell populations (LCs and dendritic epidermal T-cells: DETCs) slightly expanded rather than migrated out of the epidermis during the 24-h incubation (Figure 2B). Interestingly, de-novo TSLP could be readily detected in the culture media of 32 °C cultured WT skin explants as compared to incubation on RT (Figure 2C). In comparison, skin explants from EGFR^∆ep^ mice did not show significantly elevated TSLP production compared to WT mouse skin when cultured on 32 °C (Supplementary Figure S1). TSLP is a well-known dendritic cell activator [4]. Indeed, LCs isolated from the epidermis of the skin explants displayed enhanced expression of MHC II, CD86, and CD11c, whereas CD80 and EpCAM remained unchanged after the 24-h incubation (Figure 2D). In conclusion, WT skin explants cultured at 32 °C represent an ex-vivo model for de-novo TSLP expression and early LC activation.

### 3.3. TSLP Transcriptional Regulator NFAT Is Not Up-Regulated in EGFR^∆^^ep^ Mice, and an NFAT Pathway Inhibitor Does Not Block TSLP Expression Ex Vivo

**Figure 3:**
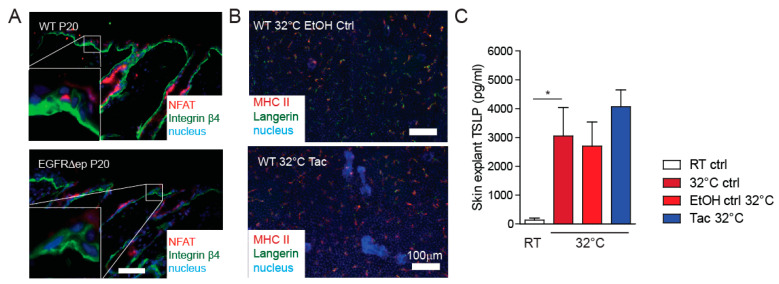
TSLP transcriptional regulator NFAT is not up-regulated in EGFR∆ep mice, and a NFAT pathway inhibitor does not block TSLP expression ex vivo. (**A**) NFAT expression was detected by immunofluorescence of 5-µm skin cryosections of WT and EGFR∆ep mice. Colours are as indicated in the pictures. (**B**) Epidermal LC network of epidermal sheets from skin explants treated with vehicle (EtOH) or tacrolimus (TAC). (**C**) Culture supernatant from WT skin explant cultures with EtOH (vehicle control) or tacrolimus (Tac) was collected after 24 h at indicated temperatures, and TSLP expression was detected by ELISA. Data represent means ± SEM. * *p* < 0.05. Data are from at least three independent experiments. Scale bars = 100 μm.3.4. TSLP transcriptional regulator AP1 is up-regulated and active in EGFR^∆^^ep^ mice.

TSLP expression can be induced from KCs through the NFAT signalling pathway to induce itch responses [13]. In order to investigate the involvement of this pathway in TSLP expression, we first analysed NFAT expression in skin sections of EGFR^Δep^ mice. High expression of NFAT could be observed in both WT control and EGFR^Δep^ skin in the immune cell compartment, with more immune cells being present in the inflamed skin of the EGFR^Δep^ mice. However, no marked up-regulation of NFAT could be observed in the KCs from EGFR^Δep^ mice (Figure 3A). Furthermore, the well-known clinically relevant NFAT inhibitor tacrolimus neither inhibits LC-specific MHC II up-regulation nor alters TSLP expression from skin explants cultured at 32 °C (Figure 3C).

This indicates no major involvement of this pathway regarding TSLP expression during early skin barrier defects in this skin explant model.

**Figure 4:**
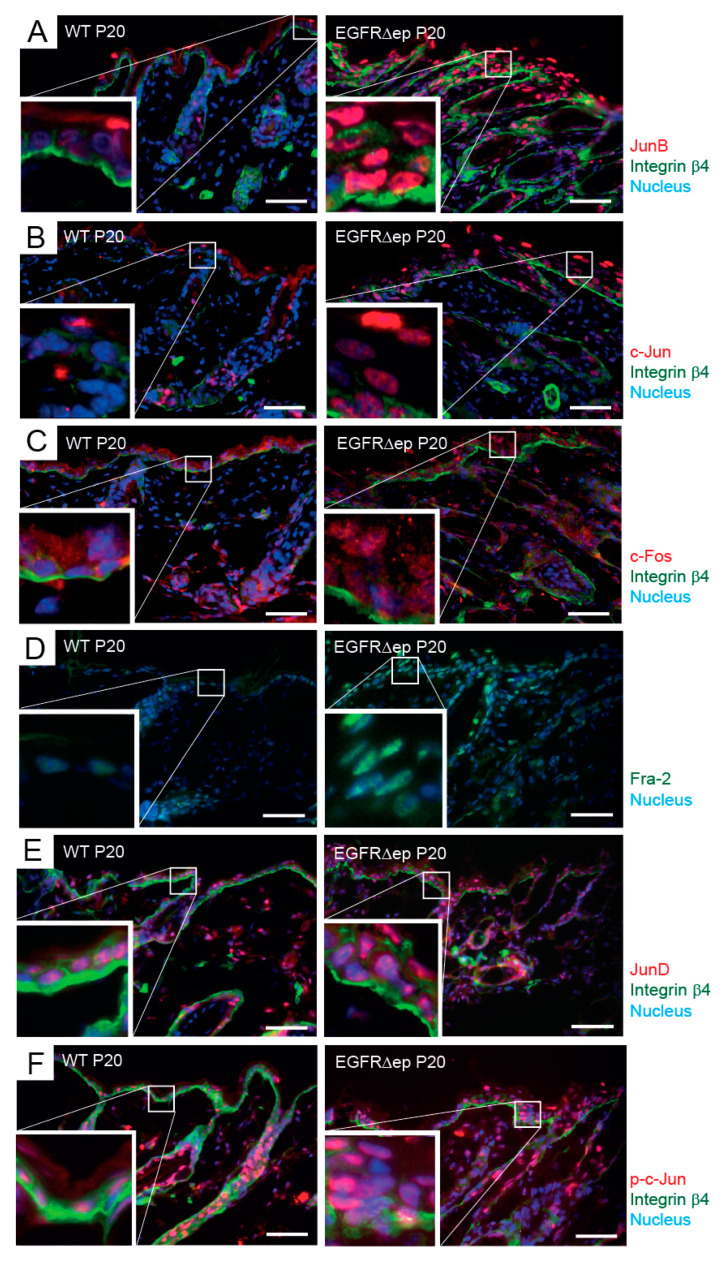
TSLP transcriptional regulator AP1 is up-regulated and active in EGFR∆ep mice. (**A**–**F**) Immunofluorescence of 5-µm back-skin cryo-sections of 20-day-old (P20) WT and EGFR∆ep mice for AP-1 members (JunB (**A**), c-Jun (**B**), c-Fos (**C**), Fra2 (**D**), JunD (**E**), and phospho c-Jun (**F**)). The basal membrane, separating epidermis (upper part) from dermis (lower part), was visualized with Integrin b4 when indicated. Nuclei were visualized with Hoechst. Colours are as indicated next to the pictures. Data are representative of three independent mice per genotype. The insets show an enlarged view of the framed epidermal areas with KCs. Scale bars, 50 µm. Mice were analysed 20 days after birth (P20).

TSLP expression can also be induced via the Activator protein-1 (AP-1) family of transcription factors [14]. In patients with atopic dermatitis, higher levels of basal AP-1 expression are found together with elevated TSLP expression, which maintains a TH2-polarised inflammatory milieu [7]. Similar to the NFAT approach, we first analysed AP-1 member expression in skin sections of EGFR^Δep^ mice and found a strong up-regulation of JunB, c-Jun, c-Fos, Fra2, and JunD in epidermal KCs as compared to WT control skin (Figure 4A–E). Phosphorylation of one of the AP-1 members, c-Jun, further indicates the activated status of this transcription factor family (Figure 4F).

These data indicate that, similar to atopic dermatitis, the AP-1 transcription factor family is up-regulated during EGFR-dependent skin inflammation.

### 3.4. JNK Pathway Inhibitor Blocks TSLP Expression Ex Vivo

In order to investigate the involvement of the AP-1 member c-Jun during the induced expression of TSLP from skin explants on 32 °C, we floated ear skin from c-Jun epidermal KO mice with no detectable KC-specific c-Jun expression (K5-cre c-Jun fl/fl, Supplementary Figure S2A). We observed no change in TSLP expression when compared to WT control skin explants at 32 °C, indicating no major involvement of c-Jun alone (Supplementary Figure S2B).

**Figure 5:**
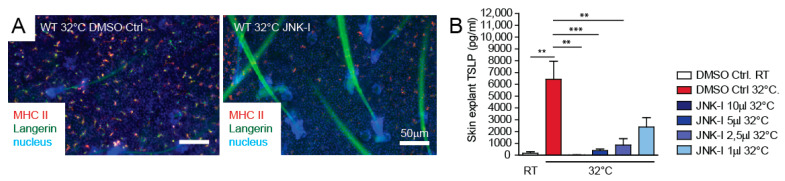
JNK pathway inhibitor blocks TSLP expression ex vivo. (**A**) Ears from 1–2-month-old WT mice were separated and floated for 24h on medium at room temperature and 32 °C. DMSO (vehicle) or JNK-Inhibitor (JNK-Inh) was added at different concentration as indicated (1 μL = 25 mM). Epidermal sheets were isolated and stained for Langerin and MHC II (I-A/I-E) to detect the activation status of the LCs. Pictures are representatives from at least three independent experiments. (**B**) Culture supernatant from skin explant cultures with DMSO (vehicle control) and JNK-Inh at indicated concentrations was collected after 24 h, and TSLP expression was detected by ELISA. Data represent means ± SEM. ** *p* < 0.01; *** *p* < 0.001. Data are from at least three independent experiments.

The JNK signalling pathway is responsible for AP-1 transcriptional activation [9]. We next tested if we could influence skin barrier immunity and TSLP expression via the inhibition of the upstream JNK pathway in ex-vivo mouse skin explants. The well-described JNK inhibitor SP600125 was able to block MHC II up-regulation on the LCs and blunted TSLP expression from skin explants cultured at 32 °C in a dose-dependent manner (Figure 5A,B).

Therefore, our data indicate that the dominant driver of TSLP expression during skin barrier defects in explant cultures is the JNK-AP1 axis.

## 4. Discussion

In this study, we showed that skin explants cultured at 32 °C represent a model for early pathogenesis during skin barrier breakdown and the subsequent immunological cascade. We demonstrated the production and secretion of TSLP in parallel to in-situ LC activation. Furthermore, TSLP expression could be dampened by inhibition of the JNK signalling pathway. This implicates a therapeutic anchor point for AD- and EGFR-I-induced adverse events. The c-Jun^Δep^ mouse skin explants, however, displayed an unaltered TSLP expression, which indicates that various members of the AP-1 family might be responsible for the TSLP induction. Indeed, TSLP expression during atopic inflammation has also been linked to c-Fos [14].

JNK1 has recently been shown to negatively control antifungal immunity, and its inhibition exerted anti-fungal effects [15]. The anti-inflammatory properties of JNK-inhibitors have also been described for BRAF-inhibitor-induced skin inflammation in vivo [16]. This condition relates to the adverse events of EGFR-Is, as we recently showed that EGFR controls the RAF-MEK-ERK cascade in KCs [10]. MEK inhibitors are known inducers of similar adverse events, as can be seen with EGFR-I during specific anti-tumour treatment [17]. Transgenic over-expression of Son of Sevenless (SOS) in the EGFR-deficient background reinstalled an active ERK signalling and prevented the barrier disintegration and subsequent TSLP production [10]. Using mice bred under germ-free conditions and mice treated with broad spectrum antibiotics, we could establish the microbiota-independent inflammatory signature, which includes TSLP expression and secretion [10]. Thereby, we can speculate that TSLP production is initiated upon the breakdown of the epidermal barrier independent of bacterial involvement. JNK activation is controlled by RAF1 to confine TSLP expression and allergic skin inflammation [16]. Our skin explant model can mimic this situation and provides a fast tool to specify the events following barrier insults.

TSLP expression from KCs was recently linked to the NFAT signalling pathway and chronic itch behaviour [13]. However, in our study, NFAT seems to be not involved in regulating LC activation and TSLP expression, as the calcineurin-blocker tacrolimus did not impact TSLP expression.

High levels of TSLP can be found in blood and skin of AD patients, and transgenic overexpression in mice leads to spontaneous, AD-like dermatitis [6,18]. It is intriguing to presume that skin explants incubated at 32 °C represent an early AD-like disease model, which enables the analysis of cytokine and chemokine expression cascades; test the involvement of different signalling pathways; and implement novel therapeutic approaches. Apart from the stress-induced reactions of the KC layer, the activation of LCs and various resident dermal immune cell population might also be examined. LCs up-regulate MHC-II and CD86, which primes them for antigen presentation. This observation is supported by the emigration of LCs during longer skin explant incubation [19,20,21]. This LC emigration model is regarded to mimic steady-state migration. Our study challenges this view by highlighting the Th2 cytokine TSLP. LC emigration from skin explant cultures during KC-specific TSLP secretion might rather reflect the situation of an early AD flare.

Skin explants can also be activated by extrinsic cytokines or stressors added to the medium or directly on the skin in order to mimic specific inflammatory situations [22]. We could recently show that nickel (NiSO_2_), when supplemented to human skin explant cultures, is able to activate the receptor tyrosine kinase Axl and leads to an up-regulation of its family member Mer on LCs [2]. They belong to the TAM family of receptors and facilitate the uptake of apoptotic cells together with the blunting of pro-inflammatory cytokine production by antigen-presenting cells [23]. We can further show that this affects LC function and leads to an exaggerated allergic skin inflammation in vivo [2].

## 5. Conclusions

Taken together, our study findings underline the importance of cultured skin explants as a model system and demonstrate that the JNK-TSLP signalling axis is among the first active immune cascades in the epidermal barrier.

## Figures and Tables

**Table 1:** Antibody resources. Cat# refers to catalogue number.

Antibodies	SOURCE	IDENTIFIER
Armenian hamster anti-mouse TCR γ/δ Antibody, FITC	Biolegend	Cat# 118106, RRID:AB_313830
Rat anti-mouse/human CD11b Antibody, Pacific Blue	Biolegend	Cat# 101224, RRID:AB_755986
Armenian hamster anti-mouse CD11c Antibody, PE/Cy5	Biolegend	Cat# 117316, RRID:AB_493566
Rat anti-mouse I-A/I-E Antibody, APC/Cy7	Biolegend	Cat# 107628, RRID:AB_2069377
Rat anti-mouse CD45 Antibody, APC	Biolegend	Cat# 103112, RRID:AB_312977
Rat anti-Langerin/CD207 Antibody (929F3.01), Alexa Fluor 488	Dendritics	Cat# DDX0362A488, RRID:AB_1148740
Rat anti-mouse I-A/I-E Antibody, PE	Biolegend	Cat# 107608, RRID:AB_313323
Rat anti-mouse/human Langerin/CD207, Alexa Fluor 488	Dendritics	Cat# DDX0362 929F3.01
Rat anti-mouse EpCAM/CD326, FITC	Biolegend	Cat# 118208, RRID:AB_1134107
Rat anti-mouse CD45, PE	Biolegend	Cat# 103106, RRID:AB_312971
Rat anti-mouse CD45, APC	Biolegend	Cat# 103112, RRID:AB_312977
Anti-beta III Tubulin Antibody (TUJ-1)	abcam	ab14545
JunD (329)	Santa cruz	sc-74
NFATc1	Santa cruz-sc-7294	7A6
Fra2 CN10	home made	
Phospho-cJun Ser73	Cell Signalling	D47G9
cJun	Cell Signalling	60A8
JunB	Cell Signalling	C37F9
cFos	THP-Medical Products	Y075085
CD104-Integrin β4 chain	BD Pharmingen™	Cat# 553745
Donkey anti-rabbit 594/488 Alexa Fluor	Invitrogen-Life Technologies	Cat# A21206
Donkey anti-rat 594/488 Alexa Fluor	Invitrogen-Life Technologies	Cat# 21208
Donkey anti-Guinea Pig 594 Alexa Fluor	Invitrogen-Life Technologies	Cat# A11076

## Data Availability

The data supporting the findings of this study are available from the corresponding author upon reasonable request.

## Supplementary Materials

The following are available online at https://www.mdpi.com/article/10.3390/life11111237/s1, Figure S1: TSLP expression is released from EGFR∆ep skin explant cultures. Figure S2: cJun is not responsible for TSLP up-regulation from ex vivo skin explants.

## References

[1] Chen Y.E., Fischbach M.A., Belkaid Y. (2018). Skin microbiota-host interactions. Nature.

[2] Bauer T., Zagórska A., Jurkin J., Yasmin N., Köffel R., Richter S., Gesslbauer B., Lemke G., StrobL H. (2012). Identification of Axl as a downstream effector of TGF-beta1 during Langerhans cell differentiation and epidermal homeostasis. J. Exp. Med..

[3] Lichtenberger B.M., Gerber P.A., Holcmann M., Buhren B.A., Amberg N., Smolle V., Schrumpf H., Boelke E., Ansari P., Mackenzie C. (2013). Epidermal EGFR Controls Cutaneous Host Defense and Prevents Inflammation. Sci. Transl. Med..

[4] Corren J., Ziegler S.F. (2019). TSLP: From allergy to cancer. Nat. Immunol..

[5] Ebner S., Nguyen V.A., Forstner M., Wang Y.-H., Wolfram D., Liu Y.-J., Romani N. (2007). Thymic stromal lymphopoietin converts human epidermal Langerhans cells into antigen-presenting cells that induce proallergic T cells. J. Allergy Clin. Immunol..

[6] Weidinger S., Beck L.A., Bieber T., Kabashima K., Irvine A.D. (2018). Nature Reviews Disease Primers Atopic dermatitis. Nat. Rev. Dis. Primers.

[7] Pastore S., Giustizieri M.L., Mascia F., Giannetti A., Kaushansky K., Girolomoni G. (2000). Dysregulated Activation of Activator Protein 1 in Keratinocytes of Atopic Dermatitis Patients with Enhanced Expression of Granulocyte/Macrophage-Colony Stimulating Factor. J. Investig. Dermatol..

[8] Zenz R., Eferl R., Kenner L., Florin L., Hummerich L., Mehic D., Scheuch H., Angel P., Tschachler E., Wagner E.F. (2005). Psoriasis-like skin disease and arthritis caused by inducible epidermal deletion of Jun proteins. Nature.

[9] Novoszel P., Holcmann M., Stulnig G., De Sa Fernandes C., Zyulina V., Borek I., Linder M., Bogusch A., Drobits B., Bauer T. (2021). Psoriatic skin inflammation is promoted by c-Jun/AP-1-dependent CCL2 and IL-23 expression in dendritic cells. EMBO Mol. Med..

[10] Klufa J., Bauer T., Hanson B., Herbold C., Starkl P., Lichtenberger B., Srutkova D., Schulz D., Vujic I., Mohr T. (2019). Hair eruption initiates and commensal skin microbiota aggravate adverse events of anti-EGFR therapy. Sci. Transl. Med..

[11] Sigismund S., Avanzato D., Lanzetti L. (2018). Emerging functions of the EGFR in cancer. Mol. Oncol..

[12] Holcmann M., Sibilia M. (2015). Mechanisms underlying skin disorders induced by EGFR inhibitors. Mol. Cell. Oncol..

[13] Wilson S.R., Thé L., Batia L.M., Beattie K., Katibah G.E., McClain S.P., Pellegrino M., Estandian D.M., Bautista D.M. (2013). The Epithelial Cell-Derived Atopic Dermatitis Cytokine TSLP Activates Neurons to Induce Itch. Cell.

[14] Murthy A., Shao Y.W., Narala S.R., Molyneux S., Zuniga-Pflucker J.C., Khokha R. (2012). Notch Activation by the Metalloproteinase ADAM17 Regulates Myeloproliferation and Atopic Barrier Immunity by Suppressing Epithelial Cytokine Synthesis. Immunity.

[15] Zhao X., Guo Y., Jiang C., Chang Q., Zhang S., Luo T., Zhang B., Jia X., Hung M.-C., Dong C. (2017). JNK1 negatively controls antifungal innate immunity by suppressing CD23 expression. Nat. Med..

[16] Raguz J., Jeric I., Niault T., Nowacka J.D., Kuzet S.E., Rupp C., Fischer I., Biggi S., Borsello T., Baccarini M. (2016). Epidermal RAF prevents allergic skin disease. eLife.

[17] A Ascierto P., Schadendorf D., Berking C., Agarwala S.S., Van Herpen C.M., Queirolo P., Blank C.U., Hauschild A., Beck J.T., St-Pierre A. (2013). MEK162 for patients with advanced melanoma harbouring NRAS or Val600 BRAF mutations: A non-randomised, open-label phase 2 study. Lancet Oncol..

[18] Yoo J., Omori M., Gyarmati D., Zhou B., Aye T., Brewer A., Comeau M.R., Campbell D.J., Ziegler S.F. (2005). Spontaneous atopic dermatitis in mice expressing an inducible thymic stromal lymphopoietin transgene specifically in the skin. J. Exp. Med..

[19] Ratzinger G., Stoitzner P., Ebner S., Lutz M.B., Layton G.T., Rainer C., Senior R.M., Shipley J.M., Fritsch P., Schuler G. (2002). Matrix Metalloproteinases 9 and 2 Are Necessary for the Migration of Langerhans Cells and Dermal Dendritic Cells from Human and Murine Skin. J. Immunol..

[20] Stoitzner P., Zanella M., Ortner U., Lukas M., Tagwerker A., Janke K., Lutz M.B., Schuler G., Echtenacher B., Ryffel B. (1999). Migration of langerhans cells and dermal dendritic cells in skin organ cultures: Augmentation by TNF-alpha and IL-1beta. J. Leukoc. Biol..

[21] Weinlich G., Heine M., Stössel H., Zanella M., Stoitzner P., Ortner U., Smolle J., Koch F., Sepp N.T., Schuler G. (1998). Entry into afferent lymphatics and maturation in situ of migrating murine cutaneous dendritic cells. J. Investig. Dermatol..

[22] Eberlin S., Silva M.S.D., Facchini G., Silva G.H.D., Pinheiro A.L.T.A., Eberlin S., Pinheiro A.D.S. (2020). The Ex Vivo Skin Model as an Alternative Tool for the Efficacy and Safety Evaluation of Topical Products. Altern. Lab. Anim..

[23] Lemke G. (2013). Biology of the TAM receptors. Cold Spring Harb. Perspect. Biol..

