# Supplementary material for: Ex-Vivo Skin Explant Culture Is a Model for TSLP-Mediated Skin Barrier Immunity
Source: Life (Basel). 2021 Nov 16;11(11):1237. doi: 10.3390/life11111237 (PMC8623134; doi:10.3390/life11111237)
Supplement: Supplementary file 1 [file life-11-01237-s001.zip › life-1442045-supplementary.pdf]

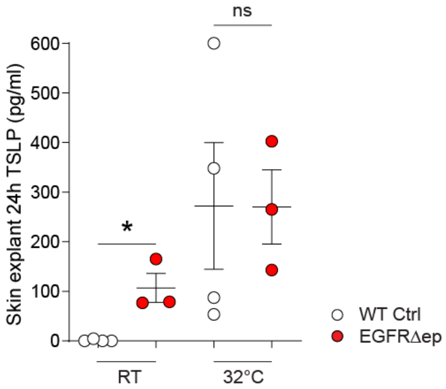

Figure S1. TSLP expression is released from EGFRΔep skin explant cultures. Skin explants from WT and EGFRΔep mice were cultured for 24 h on RT or 32 °C and TSLP was measured in the culture medium by Elisa. Data represent means ±SEM; * $p < 0{,}05$. Data are from at least three independent experiments.

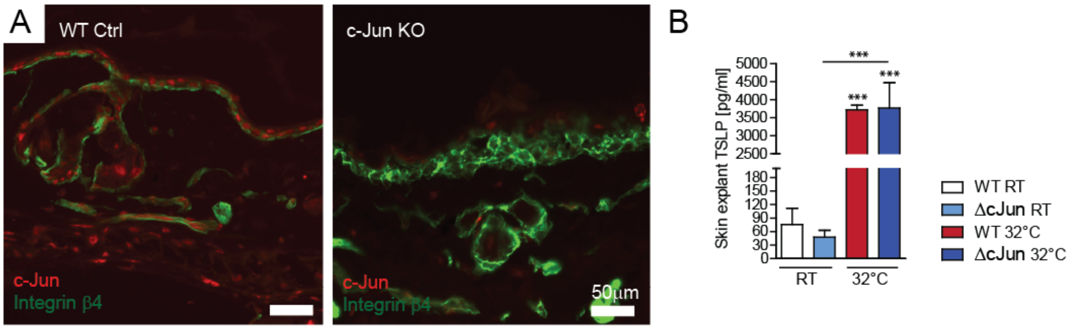

Figure S2. cJun is not responsible for TSLP up-regulation from ex vivo skin explants. (**A**) Immunofluorescence staining of cJun in ear skin cryo-sections of WT and c-Jun$^{\Delta ep}$ mice. Colours are as indicated in the pictures. (**B**) Culture supernatant from skin explant cultures of WT and cjun$^{\Delta ep}$ (c-Jun KO), respectively, was collected after 24 h and TSLP expression was detected by ELISA. Data represent means ±SEM; *** $p < 0{,}001$; Data are from at least three independent experiments.
